# Fatigue and Sleep Disturbance among Breast Cancer Patients during Treatment in Saudi Arabia

**DOI:** 10.1155/2022/1832346

**Published:** 2022-09-05

**Authors:** Fatmah Alsharif, Faygah Shibily, Wedad Almutairi, Ahlam Alsaedi, Tahani Alsubaie, Bashayer Alshuaibi, Arwa Turkistani

**Affiliations:** ^1^Medical Surgical Nursing Department, King Abdulaziz University, Jeddah 21589, Saudi Arabia; ^2^Maternity and Pediatric Nursing Department, King Abdulaziz University, Jeddah 21589, Saudi Arabia; ^3^Nursing, King Abdulaziz University, Jeddah 21589, Saudi Arabia

## Abstract

**Background:**

Fatigue and sleep disturbances are highly prevalent side effects encountered during treatment by patients with breast cancer, and they affect women's quality of life. Most studies investigating sleep and exhaustion in cancer patients provide evidence that supports a strong connection between different sleep parameters and fatigue associated with cancer.

**Objective:**

This study was to assess the level of fatigue and sleep disturbance in breast cancer patients during treatment in Saudi Arabia.

**Method:**

A descriptive cross-sectional study design was conducted on breast cancer patients undergoing treatment in Saudi Arabia. The data were collected through convenience sampling of patients. The study involved self-administered questionnaires comprising three categories: demographic data, perceived Pittsburgh Sleep Quality Index (PSQI), and Functional Assessment of Chronic Illness Therapy–Fatigue (FACIT-F). A total of 101 participants took part in this study. The significant statistical test was determined at a 95% confidence interval and at *p* < 0.05.

**Results:**

Half of the study participants were aged between 30 and 50 years. Significant differences were found in fatigue and sleep disturbance among BC patients during treatment in Saudi Arabia. A high prevalence of fatigue was found at 21.8%, and 5% of participants experienced sleep disturbance.

**Conclusions:**

Breast cancer patients in Saudi Arabia have a low overall global quality of life. The patients experience poor sleep quality and fatigue, which suggests that during treatment, patients need to be assessed routinely for these symptoms to prevent or reduce fatigue and sleep disturbance. Sleep disturbance might be correlated with fatigue.

## 1. Introduction

Worldwide, the most prevalent type of cancer is breast cancer (BC), leading to increased mortality among women [1]. It is estimated that more than one million females have diagnosed annually with BC [2]. There were 627,000 female deaths from BC in 2018, which comprised nearly 15% of all female cancer-related deaths [2]. The predominance of BC in Saudi Arabia's female population is 25.9%, and the mortality rate is 18.2% [3]. The second most common malignancy in Saudi females is BC, with a prevalence of 21.8% [4]. A cancer mortality survey among SA females has shown that BC is the 9th most common cause of death [4].

In SA, BC rates are expected to rise following several generations as the community increases and ages [4]. This disease ranks first among SA's female malignancies, accounting for nearly 22% of cases newly diagnosed with cancer [3]. In most nations, the rate of incidence of BC is also rising [3]. The mortality rate for BC was 5 times greater than that for patients with stage I tumor (credible interval) and 3.7 times greater than that for patients with stage II tumor in patients whose cancers were graded as stage IV [4].

In Western countries, the median onset age for BC patients is 65 years. However, in Saudi women, the median age for BC onset is 48 years [3]. BC is therefore a major concern for the SA population [3]. Anders and colleagues found that around 7% of women in the USA are diagnosed with BC before the age of 40, and for these women, survival rates are worse than those diagnosed at older ages [4]. Among young Saudi women, early diagnosis is a significant problem [4]. One study has shown from a logistic regression model that low BC screening use is significantly correlated with a woman's age, educational status, family income, use of hormonal contraceptives, and a positive history of previous BC [5].

During cancer care trajectory, individuals with cancer frequently struggle with different complicated symptoms that can interfere with the QOL and daily activities [1]. BC patients are exposed to several psychological and physical symptoms, which affect the quality and amount of sleep and fatigue [6]. Fatigue and sleep disturbances are highly prevalent and are encountered during treatment by patients with BC [7].

This topic has been studied in many countries, but there has been no focus specifically on BC patients [6]. Due to there being insufficient information and details in relation to BC, the authors of this paper wanted to conduct this research [6]. The majority of research examining sleep and exhaustion in cancer patients provides evidence that supports a clear association between cancer-related fatigue (CRF) and different parameters of sleep [7].

The majority of research has been performed in respect of patients with different cancers undergoing radiotherapy, chemotherapy, hormonal therapy, and surgery, for which associations have also been reported between fatigue and sleep disorders [8]. The prevalence of sleep disruption was 40% [9].

However, the QOL associated with well-being is now recognized as a virtual endpoint for cancer patients [6]. It has been shown that measuring cancer patients' QOL could enhance care and function as a prognostic factor and medical parameter [6]. The aim of the study was to assess the level of fatigue and sleep disturbance in breast cancer patients during treatment in Saudi Arabia.

The high prevalence of symptoms in the patient with BC during treatment are fatigue and sleep disturbances [7]. Roscoe et al. found that sleep disturbances are a common issue for cancer patients and survivors due to their complicated CRF relationship [8]. Considering what we know about the possible correlation between CRF and sleep disruptions, therapies that target disordered sleep and daytime sleepiness may be promising CRF treatments [8]. Given recent evidence that sleep disruption is normal in cancer patients and that it can be both a cause of and a consequence of exhaustion, it stands to reason that treating one symptom while avoiding the other may have a positive effect on the other [8].

Another study found that changes in CRF over time were significantly associated with parallel changes in nausea intensity (*r* = 0.41; *p*0.0001) and disrupted sleep (*r* = 0.20; *p*0.0001), according to Pearson correlations [10]. A further study reported that sleep disruption was found to be common in 40% of the population. A total of 154 people had sleep disturbances [9] and were tired (49% *vs*. 23%, *pb*0.001) [9]. Only the exhaustion-adjusted odds ratio (AOR) (1.90, 95% (CI) 1.10–3.30, *p*=0.020) was found to be independently correlated with sleep disruption in a multivariable analysis [9].

Imanian et al. found that Spearman correlation coefficient results indicated that fatigue and sleep quality have a major relationship (0.210) [11]. In addition, Whisenant et al. found that three fatigue grades were specified: mild decreasing (59% cycle 2, 64% cycle 3), low moderate decreasing (30% cycle 2, 25% cycle 3), and high moderate decreasing (11% both cycles) [12]. Two disturbed sleep grades were specified: mild decreasing (89% cycle 2, 81% cycle 3) and increasing (11% cycle 2, 19% cycle 3) [12]. Women in the high moderate decreasing fatigue grade were more likely to have received doxorubicin (*p*=0.02) and spent more hours lying down (*p*=0.02) [12].

Another study indicated that at T1, three groups were found (fatigued with sleep complaints, average, and minimal symptoms) and five groups were identified at T2 (severely fatigued with poor sleep, emotionally fatigued with average sleep, physically fatigued with average sleep, average, and minimal symptoms) [13]. The majority of people in a group with more severe symptoms at T1 were also in a group with more severe symptoms at T2 [13]. Group membership was strongly associated with sociodemographic/medical factors at T1 and T2 [13].

Based on the findings of Fakih et al., patients recorded poor sleep 36% of the time before chemotherapy and 58% of the time during chemotherapy [14]. Clinical insomnia was recorded by 36% of patients during chemotherapy, up from 11% before treatment [14]. This indicates a substantial symptomatic burden that is poorly documented and treated in routine clinical practice [14]. Additionally, both psychological and therapy-related factors were linked to fatigue trajectories, with psychological factors most highly linked to high fatigue at the start of and during treatment [15].

In general, higher mindfulness was linked to fewer symptoms, such as pain intensity, pain interference, fatigue, depression, anxiety, and sleep disruption [16]. The degree of association, however, varied by mindfulness facet [16]. Observing had the least frequent associations and the smallest effect sizes across symptoms, while nonreactivity, nonjudgment, and explaining had the most frequent associations and largest effect sizes [16].

## 2. Materials and Methods

### 2.1. Research Design

The work involved a descriptive cross-sectional quantitative study of BC patients of age 18 years and above who were undergoing treatment in SA.

### 2.2. Setting

Data were collected using convenience sampling of patients who received cancer treatment. The data were collected from 28 February to 29 April 2020 (for 2 months). Subjects were assessed for research eligibility and asked to complete a questionnaire to assess their fatigue and sleep disturbance symptoms. All patients received care including all treatment modalities (surgical, chemotherapy, radiation, and hormonal). Patients with BC at any stage and under active medical treatment were included.

### 2.3. Sampling and Sample Size

The data were obtained from BC patients (*n* = 101). The inclusion criteria were (1) a BC patient, (2) age of 18 years and above, (3) undergoing treatment (including chemotherapy, radiation, hormonal, or surgical), and (4) able to read Arabic. The exclusion criteria consisted of (1) a mental illness and (2) diagnosis with another type of cancer. The researchers introduced the study's aim, objectives, and significance of the study to the participants and the questionnaire took approximately 10–15 minutes to be completed. The sample size was calculated through *G* power analysis using a confidence interval of 95% and an alpha of .05. The estimated target sample size was 100 students.

### 2.4. Data Collection Method

In addition to demographic data, the data were collected using two instruments to collect data [17]. The tools are available in two languages (English and Arabic language) because the study participant is speaking the Arabic language, so the authors used the Arabic version of the tool. The first tool measures fatigue and is called the FACIT-F scale of fatigue [17]. This instrument has been checked for reliability and validity [17]. The method measures self-reported fatigue and its effect on the activities of daily life and was first introduced as a supplement to the Functional Evaluation of Cancer Therapy [17]. A subscale to the general questionnaire, the FACIT-G, comprises the FACIT-fatigue [18]. In both the general population and patients with cancer, psoriatic arthritis, rheumatoid arthritis, systemic lupus erythematosus, paroxysmal nocturnal hemoglobinuria, and Parkinson's disease, the FACIT-F scale has been validated [18]. It contains 13 questions and uses a Likert-type scale of five points (0 = not at all; 1 = *a* little bit; 2 = somewhat; 3 = quite a bit; and 4 = *a* lot). All items contribute equal weight to the total ranking [18]. The scale range is between 0 and 52, with the worst possible score being 0 and the best possible score being 52, suggesting no fatigue [18]. Each negatively worded item response is documented for this reason [18]. The FACIT-F is an important tool that can serve as a basis for enhancing our understanding of cancer patients' exhaustion [18]. Validity and reliability: The FACIT-F scale (Cronbach's alpha = 0.96) was found to have high internal validity in a 2007 report [18]. It was also found to have high reliability of test retests (ICC = 0.95) [19]. The FACIT and Fatigue Intensity Scale association was −0.799 [20].

In addition, the second tool can test the sleep disorder in patients with BC (PSQI). It was developed to determine sleep efficiency [20]. The tool involves 11 self-rated questions that consist of seven component scores: subjective sleep quality (0–3), sleep latency (0–3), sleep length (0–3), normal sleep efficiency (0–3), sleep disturbances (0–3), use of sleep medication (0–3), and daytime dysfunction (0–3) [20]. To obtain a global ranking for quality of sleep (range 0 to 21), component scores are summed up (range 0 to 3); a score greater than five indicates poor quality of sleep [20]. In other words, the higher the score, the worse is the sleep quality.

The PSQI specifies the respondents' normal sleep and waking hours, the total amount of sleep hours, the time taken to fall asleep, and other Likert-type questions [21]. The 7 clinical components denoting difficulty of sleep are then analyzed using these items: subjective sleep quality, sleep delay, sleep length, normal sleep performance, sleep disturbances, sleep medication, and daytime dysfunction [21].

The scale helps researchers assess sleep disorder over a one-month cycle by calculating a basic, global score representing the intensity of sleep disruption, according to the developers of the PSQI [21]. A few validation studies have shown that a 2- or 3-factor model, rather than the original 1-factor form, could better reflect the PSQI [21].

To assess the reliability of the PSQI-I and to create internal consistency, further studies have been carried out [22]. Cronbach's alpha for the PSQI-I was 0.72, ranging from 0.69 to 0.72 for each object [22]. There were statistically important and beneficial associations between the PSQI-I overall score and the seven component scores for the PSQI-I [22]. The correlation degree was *r* = 0.36–0.56, *p* < 0.05 for each domain. In terms of validity, the overall PSQI-I score was closely related to the overall Indo BDI-II score (*r* = 0.22, *p* < 0.05). BDI-somatic II's affective and cognitive components showed a strong association with the overall PSQI-I score (*r* = 0.17–0.19, *p* < 0.05) [22]. Sleep quality, sleep disruptions, sleep medicine, and daytime dysfunction were significantly associated with the total Indo BDI-II score (*r* = 0.11–0.25, *p* < 0.05) among the seven components PSQI-I [22].

### 2.5. Variables

A self-report questionnaire was used to collect information, and participants were approached by the researcher through electronic online invitation. The questionnaire was distributed for use via the online Google forms tool. The questionnaire contained 24 items comprising PSQI and FACIT-F. Data were obtained anonymously as responses without a request for names to be registered. Before the respondent could begin the survey, a detailed study information sheet was provided in the e-mail. Furthermore, participants were told that the collected data would be used exclusively for study purposes.

### 2.6. Data Analysis

The data were analyzed using (SPSS software) (IBM, Inc. Chicago, IL, USA) version 26. Descriptive and inferential statistics were used in this study. The descriptive statistics included frequencies and percentages. The inferential statistics used one-way analysis of variance (ANOVA), A *p*-value of <0.05 was considered statistically significant.

### 2.7. Ethical Considerations

The Nursing Faculty's Ethics and Research Committee at King Abdul-Aziz University was consulted for ethical approval of this research. Enrolment in the study was voluntary, and the collection of data was anonymous, as the participants were not requested to include their names. The research details were included on the beginning page of the online survey tool. Participants could then read the details before beginning the survey and determine if they wanted to continue. Informed consent was implied by the completion and submission of the survey by the participants.

## 3. Results

### 3.1. Demographic Data

There were 101 participants in this study. Half of the study participants were aged between 30 and 50 years. Most people in the study sample were female (96%). Moreover, half of the study participants were married (54.5%). In addition, 36.6% of them were employed and most of the employed participants (50.5%) had an income of less than 5000 Saudi Riyal. Most of the study participants (74.3%) were Saudi (Table 1).

### 3.2. Differences in Levels of Fatigue and Sleep Disturbance

In this study, we tested the differences in levels of fatigue and sleep disturbance among BC patients during treatment in Saudi Arabia related to demographic variables, which included age, gender, marital status, income, occupation, and nationality. The following paragraphs present the significant results in Table 2.

#### 3.2.1. Age

There was found to be a significant difference in daytime dysfunction among BC patients during treatment in Saudi Arabia related to age variables, for which the *p*-value of the test was 0.027. The mean and standard deviation for daytime dysfunction in the under-30 age group were 1.42 and 0.55, respectively; from 30 to 50 years, the values were 1.02 and 0.79, respectively; and for the over-50 age group, the values were 1.38 and 0.77, respectively (Table 2).

#### 3.2.2. Gender

Similarly, there was a significant difference in sleep disturbance among BC patients during treatment in Saudi Arabia related to gender variables, for which the *p*-value was less than 0.001. The mean and standard deviation for sleep disturbance and gender of females were 1.48 and 0.6, and for males, the values were 1.50 and 0.58 (Table 2).

#### 3.2.3. Marital Status

There were significant differences in sleep latency among BC patients during treatment in Saudi Arabia related to marital status variables, for which the *p*-value of the test was 0.001. The mean and standard deviation for sleep latency and marital status of married participants were 1.93 and 0.92; for single participants, values were 1.66 and 0.70; and for divorced participants, values were 1.27 and 1.35. Significant differences were found in sleep duration among BC patients during treatment in Saudi Arabia related to marital status variables, for which the *p*-value of the test was 0.001. Therefore, the mean and standard deviation for sleep duration and marital status of married participants were 1.38 and 1.04; for single participants, values were 1.48 and 0.93; for divorced participants, values were 0.55 and 0.69; and for widows, values were 3.00 and 0.00. There was found to be a significant difference in daytime dysfunction among BC patients during treatment in Saudi Arabia related to marital status variables, for which the *p*-value of the test was 0.036, or less than 5%. The mean and standard deviation for daytime dysfunction and marital status of married participants were 1.02 and 0.71; for single participants, values were 1.47 and 0.67; and for divorced participants, values were 1.36 and 0.92 (Table 2).

#### 3.2.4. Income

There were found to be significant differences in sleep latency among BC patients during treatment in Saudi Arabia related to income variables, for which the *p*-value of the test was 0.023. The mean and standard deviation for sleep latency and income group of less than 5000 Saudi Riyal were 1.84 and 0.81. Moreover, the mean and standard deviation for the group income range from 5000 to 10,000 were 1.85 and 0.97, while for the group of income range from 10,000 to 20,000 Saudi Riyal, values were 1.22 and 1.13 (Table 2). Additionally, there were significant differences in sleep efficiency among BC patients during treatment in Saudi Arabia related to income variables, for which the *p*-value of the test was 0.027. The mean and standard deviation for sleep efficiency and income group of less than 5000 were 0.84 and 1.15; for the income group range from 5000 to 10,000, values were 0.83 and 1.13; and for the income group range from 10,000 to 20,000, values were 0.64 and 1.05 (Table 2). Moreover, there was a significant difference in the use of sleep medication among BC patients during treatment in Saudi Arabia related to income variables, for which the *p*-value of the test was 0.034. The mean and standard deviation for sleep medication usage and income group of less than 5000 were 0.53 and 0.95; for the income group range from 5000 to 10,000, values were 0.69 and 1.05; and for the income group range from 10,000 to 20,000, values were 0.00 and 0.00 (Table 2).

#### 3.2.5. Employment Status

The study showed a significant difference between occupation statuses among BC patients in sleep quality, sleep efficiency, sleep medication usage, daytime dysfunction, and global score of sleep. There was a significant difference in subjective sleep quality among BC patients during treatment in Saudi Arabia related to occupation variables, for which the *p*-value of the test was 0.003. The mean and standard deviation for subjective sleep quality and the unemployed group were 1.44 and 0.96; for employees, values were 0.76 and 0.80. The mean and standard deviation for students were 1.44 and 1.05. In addition, there was a significant difference in sleep efficiency among BC patients during treatment in Saudi Arabia related to occupation variables, for which the *p*-value of the test was 0.014. The mean and standard deviation for sleep efficiency and the unemployed group were 1.06 and 1.15, while the employed group's mean and standard deviation were 0.61 and 0.96. In addition, the mean and standard deviation for students were 0.59 and 1.18. Moreover, the study showed a significant difference in sleep medication usage among BC patients during treatment in Saudi Arabia related to occupation variables, for which the *p*-value of the test was 0.044. The mean and standard deviation for use of sleep medication and the unemployed group were 0.59 and 1.10; for the employed group, values were 0.16 and 0.55; and the mean and standard deviation for students were 0.67 and 0.92. Similarly, the study presented a significant difference in daytime dysfunction among BC patients during treatment in Saudi Arabia related to occupation variables, for which the *p*-value of the test was 0.001. The mean and standard deviation for daytime dysfunction and the unemployed group were 1.18 and 0.87; for the employed group, values were 0.92 and 0.64; and for the student group, the mean and standard deviation were 1.59 and 0.50. Finally, the study sample showed a significant difference in Global PSQI Score among BC patients during treatment in Saudi Arabia related to occupation variables, for which the *p*-value of the test was 0.006. The mean and standard deviation for the Global PSQI Score and unemployed group were 9.03 and 3.66; for the employed group, values were 6.62 and 3.37; and for students, values were 8.70 and 2.74 (Table 2).

#### 3.2.6. Nationality

There was a significant difference in sleep duration among BC patients during treatment in Saudi Arabia related to nationality variables, for which the *p*-value of the test was 0.006. The mean and standard deviation for sleep duration for Saudis were 1.53 and 1.04, and for non-Saudis, values were 0.88 and 0.85 (Table 2).

#### 3.2.7. Region of Living

The study showed there was no significant difference among cancer patients with regard to the region of living.

## 4. Sleep Disturbance among Breast Cancer Patients during Treatment

### 4.1. Fatigue

In total, 21.8% of the sample study have Extreme Fatigue, 43.6% of them have quite a lot of Fatigue, 20.8% of them have Some Fatigue, and 13.9% of them have Little Fatigue.

### 4.2. Subjective Sleep Quality

In total, 26.7% of the sample study is very good at Subjective sleep quality, 44.6% of them are good at Subjective sleep quality, 14.9% of them are bad at Subjective sleep quality, and 13.9% of them are very bad at Subjective sleep quality.

### 4.3. Sleep Latency

In total, 10.9% of the sample study is very good at Sleep latency, 31.7% of them are good at Sleep latency, 32.7% of them are bad at Sleep latency, and 24.8% of them are very bad at Sleep latency.

### 4.4. Sleep Duration

In total, 25.7% of the sample study is very good at Sleep duration, 23.8% of them are good at Sleep duration, 33.7% of them are bad at Sleep duration, and 13.9% of them are very bad at Sleep duration.

### 4.5. Sleep Efficiency

In total, 53.5% of the sample study is very good at Sleep efficiency, 17.8% of them are good at Sleep efficiency, 5.9% of them are bad at Sleep efficiency, and 13.9% of them are very bad at Sleep efficiency.

### 4.6. Sleep Disturbance

In total, 1% of the sample study is very good at Sleep disturbance, 54.5% of them are good at Sleep disturbance, 39.6% of them are bad at Sleep disturbance, and 5% of them are very bad at Sleep disturbance.

### 4.7. Use of Sleep Medication

In total, 74.3% of the sample study is very good at the use of sleep medication, 14.9% of them are good at using sleep medication, 3% of them are bad at using sleep medication, and 7.9% of them are very bad at using sleep medication.

### 4.8. Daytime Dysfunction

In total, 15.8% of the sample study is very good at Daytime dysfunction, 51.5% of them are good at Daytime dysfunction, 29.7% of them are bad at Daytime dysfunction, and 3% of them are very bad at Daytime dysfunction.

## 5. Discussion

To the best of our knowledge, our study is one of the first to focus on the level of fatigue and sleep disturbance in breast cancer patients during treatment in Saudi Arabia. With an increase in cancer incidence in recent years and its impact on physical, psychological, and social dimensions, it is considered a significant health problem. As these are considered to be common side effects among BC patients. Significant differences were found in fatigue and sleep disturbance among BC patients during SA treatment. Our findings show that a high prevalence of fatigue was 21.8%. The study finding is consistent with the studies conducted previously. A study showed that the majority of patients (57.4%) reported moderate fatigue, while 21.7% and 20.9% reported severe and mild fatigue, respectively [11]. However, the study result is lower than the results reported in Norway, Italy, Germany, and Canada [23, 24] and higher than the study results found in Texas, Poland, Jordan, and India [25, 26]. This disparity may be attributed to differences in socioeconomic status and health-care delivery systems.

In our data, the participants were 101. However, the study reported 21.8% extreme fatigue, 43.6% Quite a lot of fatigue, 20.8% some fatigue, and 13.9% no or little fatigue. The similarities in these first study findings show that 115 of women, had 21.7% a severe level of fatigue [11]. Although, that second study used secondary data analysis of 548 BC females and showed a high prevalence of 75% of clinically relevant CRF in BC patients following their initial chemotherapy [10]. Therefore, due to the difference in sample size, we cannot consider that the result is accurate for generalizability. However, compared to our study the differences. In the first study, the data were collected through three questionnaires [11]. Also, in the second study, they showed the effect level of nausea directed and undirected influenced [10].

In terms of sleep disturbance, 5% of participants reported severe sleep disturbance. The sleep of breast cancer patients in this sample was characterized by reduced total sleep time, with many attributing poor sleep to pain, nocturia, feeling too hot, and coughing or snoring loudly. Thus, little attention has been paid to the role that sleep disturbance may play in contributing to this symptom. This disparity may be attributed to differences in tools that have been used to collect the data as in the present study PSQI scale, but in the other study, the author used the BFI scale. In addition, the period to collect the data was three months, but the present research has limited time.

Another study used secondary data analysis of 548 BC females and showed a high prevalence of 75% of clinically relevant CRF in BC patients following their initial chemotherapy [10]. Seventy-five percent of patients had clinically significant post-treatment CRF [10]. Linear regression showed that pretreatment CRF, more significant nausea, disturbed sleep, and younger age were significant risk factors for post-treatment CRF [10]. Path modeling showed that nausea severity influenced post-treatment CRF both directly and indirectly by influencing disturbed sleep. Similarly, both nausea severity and disrupted sleep affected post-treatment CRF directly and indirectly. According to Pearson correlations, changes in CRF were strongly associated with concurrent changes in nausea occurrence and disrupted sleep [10]. This study is similar to our demographic variables; age and marital state were significant predictors of CRF. Younger patients experienced greater CRF than did older patients. This finding is consistent with prior research, which showed that younger age was associated with higher levels of CRF in BC patients. This is most likely due to more aggressive treatments given to younger patients [10]. Moreover, there was a significant difference in gender between males and females because we studied 4 males and 97 females. Therefore, due to the difference in sample size, we cannot consider that the result is accurate for generalizability.

### 5.1. Limitations

This study had a few limitations. First, insufficient number of studies related to fatigue and sleep disturbance among breast cancer patients in Saudi Arabia. Second, the sample size is considered small which affects the generalizability of the findings. Another important limitation was not being able to collect the data face-to-face due to COVID-19 restrictions. In addition to these limitations, the time required to conduct this study was limited, which was the most significant factor for us in our study.

### 5.2. Recommendations

We recommend that future studies investigate the relationship between fatigue and sleep disturbances that will enhance the quality of life for women with breast cancer and engage a more significant number of participants by increasing the sample size of the study. However, we emphasize on collecting the data from hospital settings rather than community samples and using different data collection methods such as self-report questionnaires, medical chart reviews, and interviews such as face-to-face interviews that assure that participants to understand the survey questions well and prevent the different interpretations of our questions. Further, we suggest using different designs like a mixed-method and more advanced statistical analysis to enhance and improve the study result. Also, recommending further research could advance our scientific understanding of potential interrelationships between sleep disturbance and cancer-related fatigue and clinical interventions that help with fatigue and sleep disturbance. In addition, it increases the level of education about how to deal with fatigue and sleep disturbances in the cancer patient.

## 6. Conclusions

Breast cancer patients in Saudi Arabia had a low overall global quality of life. The patients experienced poor sleep quality and fatigue, which suggests that during treatment patients need to be assessed routinely for these symptoms to prevent or reduce fatigue and sleep disturbance. Sleep disturbance might be correlated with fatigue, and future research should investigate strategies addressing sleep disturbance and fatigue.

Both sleep and fatigue in patients with cancer provide evidence supporting a high correlation between cancer-related fatigue and various sleep parameters, including poor sleep quality, disrupted initiation and maintenance of sleep, nighttime awakening, restless sleep, and excessive daytime sleepiness.

## Figures and Tables

**Table 1:** Participants' demographic characteristics.

Variables		*N*	%
AGE	Less than 30 years	36	35.6
	From 30 to 50 years	51	50.5
	More than 50 years	13	12.9

Gender	Female	97	96
	Male	4	4

Marital status	Divorce	11	10.9
	Married	55	54.5
	Single	32	31.7
	Widow	3	3

Employment	Employee	37	36.6
	None	34	33.7
	Retired	3	3.0%
	Student	27	26.7%

Income	10,000–20,000	23	22.8%
	5000–10,000	26	25.7%
	Less than 5000	51	50.5%
	More than 20,000	1	1.0%

%: percentage. *N*: number of participants. Total study participants: 101.

**Table 2:** Statistical difference among study participants' demographic variables in sleep quality level.

Demographic variables	Sleep quality level		*N*	Mean	Std. Deviation	Std. error	95% confidence interval for mean		Minimum	Maximum	*F*	Sig.
							Lower bound	Upper bound				
Age	Daytime dysfunction	<30 years	36	1.42	0.55	0.09	1.23	1.60	0.00	2.00	3.759	0.027
		30–50 years	51	1.02	0.79	0.11	0.80	1.24	0.00	3.00		
		>50 years	13	1.38	0.77	0.21	0.92	1.85	0.00	3.00		

Marital status	Sleep latency	Married	55	1.93	0.92	0.12	1.68	2.18	0.00	3.00	5.544	0.001
		Single	32	1.66	0.70	0.12	1.40	1.91	1.00	3.00		
		Divorce	11	1.27	1.35	0.41	0.37	2.18	0.00	3.00		
		Widow	3	0.00	0.00	0.00	0.00	0.00	0.00	0.00		
	Sleep duration	Married	53	1.38	1.04	0.14	1.09	1.66	0.00	3.00	5.715	0.001
		Single	31	1.48	0.93	0.17	1.14	1.82	0.00	3.00		
		Divorce	11	0.55	0.69	0.21	-+0.08	1.01	0.00	2.00		
		Widow	3	3.00	0.00	0.00	3.00	3.00	3.00	3.00		
	Daytime dysfunction	Married	55	1.02	0.71	0.10	0.83	1.21	0.00	3.00	2.968	0.036
		Single	32	1.47	0.67	0.12	1.23	1.71	0.00	3.00		
		Divorce	11	1.36	0.92	0.28	0.74	1.98	0.00	3.00		
		Widow	3	1.00	0.00	0.00	1.00	1.00	1.00	1.00		

Employment	Subjective sleep quality	None	34	1.44	0.96	0.16	1.11	1.78	0.00	3.00	6.287	0.003
		Employee	37	0.76	0.80	0.13	0.49	1.02	0.00	3.00		
		Student	27	1.44	1.05	0.20	1.03	1.86	0.00	3.00		
	Use of sleep medication	None	34	0.59	1.10	0.19	0.20	0.97	0.00	3.00	3.230	0.044
		Employee	37	0.16	0.55	0.09	-0.02	0.35	0.00	3.00		
		Student	27	0.67	0.92	0.18	0.30	1.03	0.00	3.00		
	Daytime dysfunction	None	34	1.18	0.87	0.15	0.87	1.48	0.00	3.00	7.297	0.001
		Employee	37	0.92	0.64	0.11	0.71	1.13	0.00	3.00		
		Student	27	1.59	0.50	0.10	1.39	1.79	1.00	2.00		
	Global PSQI score	None	34	9.03	3.66	0.63	7.75	10.30	4.00	17.00	5.435	0.006
		Employee	37	6.62	3.37	0.55	5.50	7.75	1.00	15.00		
		Student	27	8.70	2.74	0.53	7.62	9.79	4.00	14.00		

Income	Sleep latency	Less than 5000	51	1.84	0.81	0.11	1.62	2.07	1.00	3.00	3.320	0.023
		5000–10,000	26	1.85	0.97	0.19	1.46	2.24	0.00	3.00		
		10,000–20,000	23	1.22	1.13	0.23	0.73	1.70	0.00	3.00		
		More than 20,000	1	3.00					3.00	3.00		

Number (*N*)/standard deviation (Std.)/frequency (*F*)/significance probability (Sig.), analysis of variance (ANOVA), *∝* < 0.05 two tailed.

## Data Availability

The datasets used for this study are available from the corresponding author upon reasonable request.
